# The autophagy interaction network of the aging model *Podospora anserina*

**DOI:** 10.1186/s12859-017-1603-2

**Published:** 2017-03-27

**Authors:** Oliver Philipp, Andrea Hamann, Heinz D. Osiewacz, Ina Koch

**Affiliations:** 1 Molecular Bioinformatics, Institute of Computer Science, Faculty of Computer Science and Mathematics and Cluster of Excellence ‘Macromolecular Complexes’, Johann Wolfgang Goethe-University Frankfurt am Main, Robert-Mayer-Str. 11-15, Frankfurt am Main, 60325 Germany; 2 Molecular Developmental Biology, Institute of Molecular Biosciences, Faculty for Biosciences & Cluster of Excellence ‘Macromolecular Complexes’, Johann Wolfgang Goethe-University Frankfurt am Main, Max-von-Laue-Str. 9, Frankfurt am Main, 60438 Germany

**Keywords:** Autophagy, Aging, *Podospora anserina*, Protein-protein interaction, ATG8, Network analysis, Topological properties

## Abstract

**Background:**

Autophagy is a conserved molecular pathway involved in the degradation and recycling of cellular components. It is active either as response to starvation or molecular damage. Evidence is emerging that autophagy plays a key role in the degradation of damaged cellular components and thereby affects aging and lifespan control. In earlier studies, it was found that autophagy in the aging model *Podospora anserina* acts as a longevity assurance mechanism. However, only little is known about the individual components controlling autophagy in this aging model. Here, we report a biochemical and bioinformatics study to detect the protein-protein interaction (PPI) network of *P. anserina* combining experimental and theoretical methods.

**Results:**

We constructed the PPI network of autophagy in *P. anserina* based on the corresponding networks of yeast and human. We integrated PaATG8 interaction partners identified in an own yeast two-hybrid analysis using ATG8 of *P. anserina* as bait. Additionally, we included age-dependent transcriptome data. The resulting network consists of 89 proteins involved in 186 interactions. We applied bioinformatics approaches to analyze the network topology and to prove that the network is not random, but exhibits biologically meaningful properties. We identified hub proteins which play an essential role in the network as well as seven putative sub-pathways, and interactions which are likely to be evolutionary conserved amongst species. We confirmed that autophagy-associated genes are significantly often up-regulated and co-expressed during aging of *P. anserina*.

**Conclusions:**

With the present study, we provide a comprehensive biological network of the autophagy pathway in *P. anserina* comprising PPI and gene expression data. It is based on computational prediction as well as experimental data. We identified sub-pathways, important hub proteins, and evolutionary conserved interactions. The network clearly illustrates the relation of autophagy to aging processes and enables further specific studies to understand autophagy and aging in *P. anserina* as well as in other systems.

**Electronic supplementary material:**

The online version of this article (doi:10.1186/s12859-017-1603-2) contains supplementary material, which is available to authorized users.

## Background

In the last decade, different forms of autophagy were detected as major pathways active in recycling during starvation and in molecular quality control (QC). During macroautophagy, hereafter termed autophagy, molecules, organelles, or whole bacteria become enclosed by membranes. The resulting autophagosomes are subsequently delivered to lysosomes, in animals, or the vacuole, in plants and fungi, where they are enzymatically degraded. The building blocks of the degraded components, e.g. amino acids, are reused to generate new functional components. The main molecular processes are conserved among organisms. A core machinery encoded by “autophagy-related genes” (ATG) controls the induction of autophagy, vesicle nucleation and expansion, fusion of autophagosomes with lysosomes or vacuoles, and the final degradation of the corresponding components [14, 21].

In various organisms, evidence for a link of autophagy with aging processes has been demonstrated [18]. For example, an impaired autophagy system shortens the lifespan of mice [36] or is involved in the development of various age-dependent diseases such as Alzheimer’s, Huntington’s, or Parkinson’s disease [27]. At the same time, an increase of autophagy by treatment with rapamycin or by overexpression of specific autophagy-related genes leads to an extension of the lifespan in several organsisms. For example, in yeast treatment with 10, 20, and 40 *nM* rapamycin extends chronological lifespan. This effect is dependent on the presence of ATG1 and ATG7 [2]. In fruit flies, 50, 200 and 400 *μ* *M* rapamycin increases median lifespan by up to about 20% in females and to a lesser extent also in males [4]. Moderate overexpression of *Atg5* results in enhanced autophagy and extends median lifespan by 17% in mice [36].

In the fungus *Podospora anserina*, first evidence for a role of autophagy in aging was obtained from a genome-wide longitudinal transcriptome analysis [34]. In this fungal aging model, growth of peripheral hyphae is limited. After about 25 days wild-type strain ’s’ stops growth and dies. The lifespan of this fungus is defined as the period of growth and can be measured in days or in centimeters [30]. In the longitudinal study, the transcriptome of *P. anserina* was captured at seven consecutive age points and analyzed. The approximately 10,000 expression profiles were filtered to yield age-dependent profiles. Two groups of expression profiles with similar patterns were of particular interest: 1,202 continuously down- and 418 up-regulated profiles. A gene ontology (GO) analysis evinced that genes involved in autophagy were significantly up-regulated (*p*-value= 3.52*e*−04) while those of the ubiquitin proteasome system (*p*-value= 9.90*e*−04) were down-regulated (the details of the analyses are described in Philipp et al. (2013) [34]).

A subsequent analysis revealed that autophagy is a longevity assurance mechanism in *P. anserina* [19]. Ablation of essential autophagy machinery components, such as ATG1, leads to a shortened lifespan of the corresponding strains. Moreover, it was found that aging of the strains leads to an increase of autophagosomes and magnified autophagy activity.

The identification of autophagy as a longevity assurance mechanism in *P. anserina* is consistent with findings proposed, but not mechanistically elucidated, in other systems [22, 23, 32]. Nevertheless, only little is known about the regulatory network of autophagy as a QC mechanism effective during aging. A systematic analysis of the involved components of the autophagy machinery during aging is a promising approach to uncover new information about these branches of the network relevant for aging and lifespan control.

Although *P. anserina* is a well-established aging model for which a large body of data about pathways, affecting aging and lifespan, has been generated [30, 41], there is only little known about the single proteins and interactions involved in autophagy in this species. For example, the KEGG database [16] gives a small sub-network of autophagy in *P. anserina*. Recently, the STRING database [11] maintains some predicted interactions for *P. anserina* but with a very limited possibility to track the source of the provided interactions.

To overcome this incompleteness of PPI data, we applied the software tool PATH2PPI [33] to predict the PPI network of autophagy in *P. anserina* based on information about autophagy from the two reference systems yeast and human. Additionally, we performed a yeast two-hybrid analysis to identify interaction partners of the *P. anserina* ATG8 protein, a central component of autophagy, which is homologous to the ATG8 protein in yeast and the LC3/GABARAP protein family in human. Combining the findings of the PPI prediction approach with those of the yeast two-hybrid analysis and the expression profiles of the age-dependent transcriptome analysis, we were able to generate a comprehensive putative PPI network of autophagy in *P. anserina*, which illustrates the relation between autophagy and aging.

## Methods

### Yeast two-hybrid analysis

Using PaATG8 (UniProt Q8J282) as a bait, a yeast two-hybrid analysis was performed by Dualsystems Biotech AG (Zurich, Switzerland). The bait construct for yeast two-hybrid screening was generated by subcloning the cDNA, encoding amino acids 1 to 115 of PaATG8 (= Pa_3_5250) into the vector pLexA-DIR (Additional file 1). To prevent conjugation to substrates, not the full-length protein sequence (121 amino acids) was used, but a truncated version, lacking the last six codons (encoding Gly-Gly-Phe-Glu-Thr-Ala). The bait construct was transformed into the NMY32 yeast strain (MATa his3200 trp1-901 leu2-3,112 (lexAop)8-ADE2 LYS2::(lexAop)4-HIS3 URA3::(lexAop)8-lacZ GAL4) using standard procedures [12]. Correct expression of the bait was verified by western blot analyses of cell extracts using a mouse monoclonal antibody directed against the LexA domain. The absence of self-activation was verified by co-transformation of the bait together with a control prey and selection on minimal medium, lacking the amino acids tryptophan, leucine, and histidine (selective medium). For the yeast two-hybrid screen, the bait was co-transformed together with a cDNA library into NMY32. To obtain a cDNA library, total RNA of three *P. anserina* wild-type isolates (strain ’s’) was isolated from liquid cultures treated with 500 ng/ml rapamycin 3.5 h before harvest. pGAD-HA was used as prey vector (Additional file 2).

5∗10^6^ transformants were screened, yielding 96 transformants that grew on selective medium and were positively tested for *β*-galactosidase activity, using a quantitative *β*-galactosidase assay. From 70 of these clones successful plasmid rescue and sequencing from a 5’ junction sequencing primer was possible. Six clones were discarded because the coding sequence was not in frame. Two additional clones were discarded since they encode transcription factors and therefore, obviously represent false positives. The remaining 62 clones were assigned to 21 different proteins (see Additional file 3).

### Databases and data repositories

In the UniProt database, we searched for proteins tagged with the key words “autophagy” or “macroautophagy” and additionally declared to be “reviewed” to get the high-quality, manually annotated entries based on experimental results [39].

To get all known interactions, we used the latest IREFINDEX MITAB file (release 14) for human (taxonomy id 9606) and yeast (taxonomy id 559292) [37]. The sets of relevant reference proteins and the IREFINDEX files were assigned to PATH2PPI. This software package uses IREFINDEX for retrieval of interaction data since it is a meta database for protein-protein interactions which combines interactions from different databases and arrange them with many additional information. This information is provided in a well compiled format and can easily be parsed and handled by computational analysis pipelines and methods.

For the protein or gene identifiers of yeast and human we used the accession numbers provided by the UniProt database. In contrast, each *P. anserina* accession number or identifier, respectively, was adopted from the *P. anserina* genome database [10].

### Homology search and settings for PATH2PPI

PATH2PPI requires the homologous relations for each of the previously gathered proteins. We performed a BLAST search, using the BLAST toolkit (release 2.2.29) with default settings and an E-value cutoff of 1*e*−4 [5].

We applied PATH2PPI, using default settings except the definition of the homology range, which is defined by a lower and an upper E-value bound, i.e. in this context we interpreted and defined the BLAST E-value as a degree of “similarity” (see Philipp et al. (2016) [33]). If a BLAST E-value for two proteins is greater than or equal to the upper bound, *h*_*u*_, the score equals 0. An E-value which is less than or equal to the lower bound, *h*_*l*_, will lead to the top score of 1. Thus, each E-value between these bounds will be scored according to the range [ 0,1]. The existence of only one homolog to a protein with an E-value of at most 1*e*−5 will also lead to the top score of 1. To find appropriate parameters for these two bounds, we repeatedly run the prediction algorithm, using different values for *h*_*u*_ and *h*_*l*_. We started with an E-value of 1*e*−20 and decrease it stepwise by 1*e*−20 until we reach 1*e*−200:

*h*_*u*_= [ 1*e*−20,1*e*−40,1*e*−60,…,1*e*−160] and

*h*_*l*_= [ *h*_*u*_∗1*e*−40,*h*_*u*_∗1*e*−60,*h*_*u*_∗1*e*−80,…,1*e*−200].

In our case, it is not feasible to use default evaluation approaches of prediction classifiers, like ROC curves or a precision and recall approach, since only sparse information is available. For example, a false-positively predicted interaction could occur due to the lack of information. To choose appropriate values for the homology range and to evaluate the different results, we compared each network with the very small autophagy network of *P. anserina* of the KEGG database [16] (see Additional file 4). This small, manually curated network consists of 15 proteins and 12 interactions, which are based on a mapping of *P. anserina* proteins and the corresponding interactions to the basal autophagy pathway. This basal pathway is strongly conserved throughout most eukaryotic organisms. We covered as much as possible of these known interactions and proteins.

### Statistical significance

In biological research, the experimental data are often incomplete and of different quality and quantity. To ensure that the set of data we consider is large enough for biological analysis, we have to prove that the model could not be randomly formed, but exhibits “real-world” properties.

Furthermore, the transfer and compilation of interactions from different sources to one PPI network must rely on well-defined rules and assumptions. The applied approach must not produce a random network. To mathematically prove the predicted network for biological consistency and to decide whether it differs from networks generated by chance, we computed several topological features [28]. We compared these features with the median value of thousand networks of randomly chosen interaction partners of the same proteins. We took two sets of randomly generated networks. The first set (*set 1*) consists of networks with unchanged node degree, i.e. the number of adjacent edges was the same, only the corresponding interacting partners were randomly changed. The second set (*set 2*) consists of randomly chosen interaction partners, only preserving the total number of edges. Which one of the two sets we considered depended on the topological feature to be compared. Unless otherwise stated, for the computation of the single topological features, we applied the functions of the IGRAPH-package [9] using default settings. We computed the topological features for the predicted and for each random network to show the differences between them. For this comparison approach, we considered the predicted proteins and interactions resulted from PATH2PPI, but not the proteins from the yeast two-hybrid analysis.

We considered the following topological features: the diameter, modules and modularity, the transitivity or clustering coefficient, the node degree and the node betweenness (hereinafter called degree or betweenness, respectively). The comparison of the different topology values of the predicted network with those of the random networks requires appropriate statistical tests. Hence, to compute empirical *p*-values for each of the considered topologies, we applied appropriate statistical tests for the corresponding topological feature (see detailed descriptions below and Additional file 5).

#### Diameter

The diameter of a network is defined as the longest path of all shortest paths between each two nodes. “Real-world” networks, such as metabolic or protein interaction networks, exhibit a small diameter, having a “small world” architecture, indicating that any node can be reached via a relatively short path from another node [44]. We compared the diameter of the predicted network with the median diameter of the randomly rewired networks (*set 1*). Since the set of diameters of the random networks represents a discrete distribution, we computed the relative frequency of each diameter which can be interpreted as an empirical *p*-value.

#### Modules and modularity

Modules are subgraphs, in which the connections within them are much denser than between them. In a biological network, modules often correspond to different functional sub-pathways. The modularity represents a quality measure for network partitioning. For module detection we applied a random walk approach as described by [35]. The computation of the modularity is based on the method of Clauset et al. (2004) [8]. To compute an empirical *p*-value for the modularity of the predicted network, we first applied the Shapiro-Wilk test on the modularities of the random networks to check whether they are normally distributed. Subsequently, we used the normal distribution with the mean and standard deviation of the random modularities (*set 1*) to compute the *p*-value of the predicted network’s modularity.

#### The transitivity or clustering coefficient

The transitivity or clustering coefficient indicates how dense the nodes of a network are connected. More precisely, the global transitivity is defined as the ratio of the triangles and the connected triples in the network. If *n* is the number of triangles and *t* the number of triplets, then the clustering coefficient *c* is computed by $c = \frac {3n}{t}$ [3]. To compute the *p*-value for the predicted network’s cluster coefficient, we applied the same test procedure as for the modularity.

#### Node degree and node betweenness

To reveal major hubs in the network, we computed the node degrees and the node betweennesses. Generally, PPI networks exhibit the scale-free property, i.e. they consist of some nodes with a high degree and many nodes with a low degree, and the degree distribution follows a power law.

The betweenness property is an indicator for the importance of a node, i.e. it can reveal how strong a node influences a network. The higher a node’s betweenness the more increases its importance. It indicates the “traffic load” on one node under the assumption that the flow of information follows the shortest path.

Each network exhibits a certain distribution of degrees and betweennesses. To compare the cumulative distribution of the random networks (*set 2*) with the distribution of the predicted network, we applied the Kolmogorov-Smirnov test, which computes a *p*-value for the probablity that two samples were drawn from the same distribution. A significantly low *p*-value will indicate that the random network’s betweennesses and degree distributions, are significantly different from those of the predicted network.

### Statistical test for co-expression and significant regulation

We applied two different approaches to integrate the information available from the corresponding age-dependent expression profiles of the longitudinal transcriptome analysis [34].

First, we computed the Pearson correlation coefficient (Pcc) for each pair of expression profiles of all genes. A pair of profiles was assumed to be co-expressed, if the Pcc is greater than or equal to a threshold of 0.9. Two profiles were assumed to be expressed in opposite directions, if the Pcc is less than or equal to −0.9. We divided these Pcc values into four groups to achieve the contingency table for Fisher’s exact test, first, a group of all co-expressed pairs of genes, second, a group which comprises all other co-expressed pairs, third, a group of all autophagy gene pairs which are not co-expressed, and fourth, a group of pairs which were neither co-expressed nor involved in autophagy. To compute expectation values or the number of expected co-expressed genes in the group of autophagy-associated genes, we applied the hypergeometric distribution function.

Second, we computed the Pcc of each expression profile in correlation with age to reveal which genes are up- or down-regulated in the course of aging. According to the statistical approach for co-expression, we applied the Fisher’s test to prove that the number of up-regulated transcripts of the predicted PPI network is higher than expected by chance. For this purpose, we divided the 10,059 expression profiles into four sets to get the contingency table, first, a group of up-regulated transcripts in the PPI network, second, all other transcripts, third, all other up-regulated profiles, and fourth, the remaining profiles.

The entire gene expression data from the age-dependent transcriptome analysis can be found in Philipp et al. (2013) [34] or is available at the European Bioinformatics Institute’s ArrayExpress public data repository (http://www.ebi.ac.uk/arrayexpress/) with the accession number E-MTAB-2016.

## Results

**Fig. 1:**
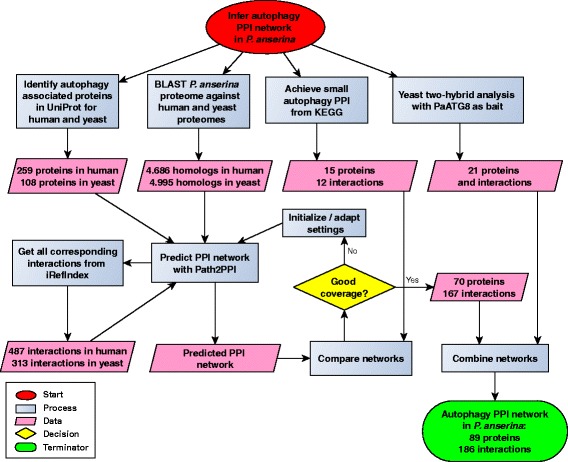
Flowchart of the analysis pipeline which was applied to infer the autophagy PPI network in *P. anserina*. At the beginning of the pipeline, each autophagy-associated protein from yeast and human was identified. In parallel, a comprehensive BLAST search was applied, using the proteomes of *P. anserina*, yeast and human. Based on the initial protein set, the software package PATH2PPI was applied to find each interaction in the IREFINDEX database which is associated with autophagy in yeast and human. Subsequently, PATH2PPI used the sets of reference proteins, interactions and BLAST results to predict the putative autophagy PPI in *P. anserina*. To achieve the best possible prediction parameters, the prediction approach was repeated several times and each result was compared with the small, manually curated autophagy PPI network from KEGG. The best predicted PPI network was combined with the experimental results of a yeast two-hybrid analysis. Finally, the pipeline led to the autophagy PPI network in *P. anserina* which consists of 89 proteins and 186 interactions

To achieve the autophagy PPI network of *P. anserina* we applied a comprehensive bioinformatics pipeline, which includes different data sets, sources and analysis approaches (see flowchart in Fig. 1).

### Initial data gathering and homology comparison

We applied the software package PATH2PPI [33] which requires three data sets for both reference species. First, PATH2PPI requires the proteins which are associated with autophagy in the corresponding species. In the UniProt database, we identified 259 proteins in human and 108 proteins in yeast directly or indirectly associated with autophagy. Second, PATH2PPI uses the IREFINDEX repository to find all known interactions between these proteins. We identified 487 interactions in human for which both proteins were in the initial protein list. Analogously, we found 313 interactions for yeast (see Additional file 6). Third, PATH2PPI needs to know the homologous relationships between each protein of *P. anserina* and each protein of human and yeast. We applied a comprehensive BLAST search, using the proteomes of *P. anserina*, yeast, and human. We identified 4686 *P. anserina* proteins with one or more human homologs and 4995 *P. anserina* proteins with yeast homologs, each with an E-value less than or equal to 1*e*−4.

### Parameter fitting for PPI network prediction

To determine appropriate initial parameters for the construction of the PPI network, we first run the prediction algorithm several times with different values for the lower and upper bound of the homology range. Subsequently, we compared the predicted PPI networks with the “small” autophagy PPI network of *P. anserina* in the KEGG database, which consists of 15 proteins and 12 interactions. The best possible result was a coverage of 14 proteins (93%) and 9 (75%) interactions (see Additional file 7). Since we wanted the best possible prediction with a minimum of strictness and a maximum of possible discrimination between the different E-values, we applied a range of [ 1*e*−200,1*e*−20] for the autophagy prediction approach.

### Comparison of autophagy-associated interactions in human, yeast, and *P. anserina*

**Fig. 2:**
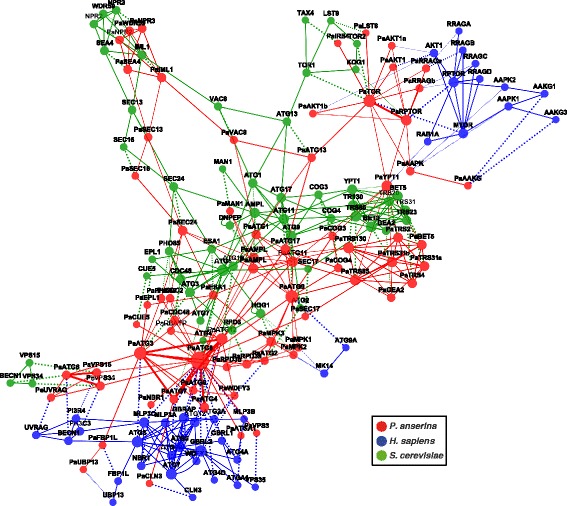
Reference and predicted PPI networks in relation with the inferred homology network of autophagy in *P. anserina*. The figure illustrates the relation of each reference network with the predicted network. This hybrid network is a combination of different network types: it comprises both reference PPI networks, the predicted PPI network and the homology network which combines each of the PPI networks. All proteins from human (*blue nodes*) and yeast (*green nodes*) and their corresponding interactions used for the *P. anserina* PPI network (*red nodes and edges*) are shown. Homologous relationships between two proteins are depicted as *dotted lines*

Figure 2 depicts the homology-based, hybrid PPI network, which consists of all proteins of the human’s and yeast’s PPI networks considered for the autophagy PPI network of *P. anserina*, with 70 proteins and 167 potential interactions (see also Additional file 8). It is based on 39 proteins and 75 interactions of the human PPI network and 50 proteins and 114 interactions of the yeast PPI network. Most of the reference interactions (see Additional file 9: Table S4) are characterized by the terms “physical interactions” (61%) or “direct interaction” (27%), in contrast to the remaining PPIs characterized by “association” (2%), “colocalization” (<2%), or “covalent binding” (<1%). Less than 10% of the reference interactions were not characterized by IREFINDEX (not shown). Additionally, each homologous relation is depicted as dotted edge. Overall, 57 homologous relationships of *P. anserina* proteins to human and 65 to yeast were detected.

The result from the initial parameter fitting approach was a first proof of concept. We compared the predicted PPI network with the “small” autophagy PPI network of *P. anserina* (see Additional file 4) and found that 14 of the 15 proteins and 9 of the 12 interactions were correctly predicted. The protein Pa_1_7190 (putative ATG10, as referred by the KEGG database) was not found in the predicted network, since no homolog exists neither in yeast nor in human. The assignment of the protein Pa_1_7190 to ATG10 by KEGG was most probably based on a homologous protein in a closely related fungus, for which the ATG10 protein had already been investigated and characterized. Two of the three not predicted interactions are based on Pa_1_7190. The third one is the interaction of Pa_2_1770 (putative PaATG7) with Pa_5_5430 (putative PaATG4). In the predicted PPI network, both proteins are not directly interacting, but are connected via one of the three proteins, Pa_3_5250 (PaATG8), Pa_1_20610 (PaATG3), or Pa_4_7460 (PaATG12). The missing direct link between PaATG7 and PaATG4 was probably due to the very strict criteria and settings we applied for the network prediction approach. This ensured that we avoid as much false positives as possible but could also miss potential true positives.

### Yeast two-hybrid analysis

The yeast two-hybrid analysis, using PaATG8 as a bait, revealed 21 putative interacting partners of PaATG8. Additional file 10: Figure S1 (red nodes) depicts the corresponding interactions and all homologous proteins from yeast and human with an E-value of at least 1*e*−20. Interestingly, we found interactions in the reference species of ATG8 with ATG4 and of ATG8 with NBR1. These two interactions were the most promising ones provided by the yeast two-hybrid analysis because independent clones were found seven and 26 times, respectively (see numbers at the red edges in Additional file 10: Figure S1).

### The autophagy PPI network

**Fig. 3:**
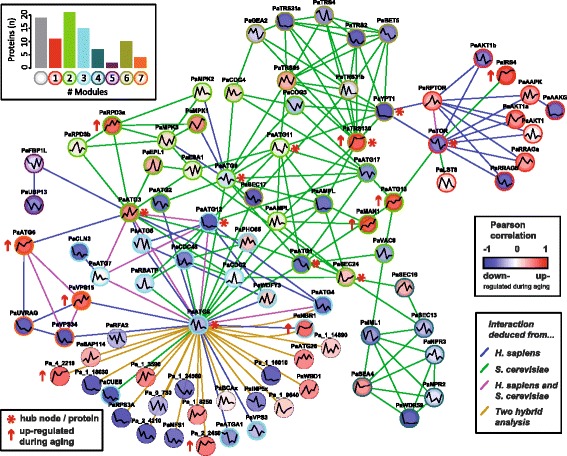
Predicted autophagy PPI network of *P. anserina* with age-dependent expression profiles. The colors of the solid lines indicate the species that exhibits the interaction (*green edges* for yeast and *blue* for human). If an interaction was found in both species, the edge is colored *purple*. In addition, if an interaction was found in the yeast two-hybrid analysis, an edge is colored *yellow*. For example, an interaction of PaATG8 and PaATG4 was found in yeast and human (*purple edge*) and additionally in the yeast two-hybrid analysis (*yellow*). Hence, this interaction is represented by two edges (*purple* and *yellow*), since it was found in all three PPI data sources. The nodes are color-coded according to the Pearson correlation coefficient of the corresponding age-dependent expression profiles. The more the expression of a transcript was increased during aging the more red is the node. The opposite direction is colored *blue*. The *red arrows* indicate transcripts which were continuously up-regulated during aging according to [34]. Red stars highlight the hub nodes, see Table 1. The number of proteins per module - from module 1 (*red*) to module 7 (*orange*) - are depicted in the bar plot in the upper left corner. The *grey bar* corresponds to proteins which were not assigned to a module, but subsequently added. Nodes, which were grouped to one of the seven functional modules, were discriminated by similar border colors

We combined the two PPI networks, the predicted and the one deduced from the yeast two-hybrid analysis, to one autophagy PPI network of *P. anserina* (Fig. 3). The PPI network consists of 89 proteins participating in 188 interactions (see also Additional file 9). Additionally, we integrated the age-dependent expression profiles of the corresponding transcripts from the former transcriptome study to indicate which of the autophagy-related genes are down- or up-regulated during aging [34].

**Table 1:** Results of the topological analysis

Topology	Pred. net.	Rand. net.	Test statistic	*p*-value
	(median)			
Diameter	8	6	Relative frequencies	0.007
Modularity	0.57	0.32	Shapiro-Wilk + Norm.dist	1.22*e*−13
Transitivity	0.35	0.12	Shapiro-Wilk + Norm.dist.	2.74*e*−42
Betweenness	*NA*	*NA*	Kolmogorov-Smirnov	1.17*e*−08
Degrees	*NA*	*NA*	Kolmogorov-Smirnov	0.09

Different test procedures were applied according to the corresponding topological feature (see “Methods” section and Additional file 5). The topological values of the predicted network and the median values of the random networks are listed. In addition, the corresponding *p*-values are shown, indicating the significance of the difference between the random and the predicted network. The computation of a single topological value for transitivity and betweenness was not applicable, since each node of each network has one betweenness and one degree. Here, only the distributions had been compared using the Kolmogorov-Smirnoff test

Many of the predicted proteins were unknown or uncharacterized for *P. anserina* and no trivial protein names were assigned. Hence, if applicable, we consider the protein names of the most homologous proteins from yeast or human, respectively, otherwise we used the protein identifiers provided by the *P. anserina* genome database [10].

### The global topology of the PPI network indicates biological significance

Biological networks differ from randomly generated networks by well-defined topological features, such as the reachability of the single nodes, the degree of cross-linking and grouping, and the general size of the network. To substantiate the biological significance of the elaborated network, we computed different topology features. These features were compared with those of the randomly generated networks (see Table 1 and Additional file 5). The first topological feature was the network’s **diameter**. The median diameter of the random networks was 6 and of the predicted network 8 (*p*-value=0.007). As one may assume that biological networks have smaller diameters due to the need of increasing the network’s efficiency, e.g. transition time or the flow of information, the result seems to be counterintuitive. Nevertheless, biological networks have diameters greater than expected, since a higher degree of modularization of a network increases its diameter [43].

We compared the **modularities** of the random networks and the predicted network. We detected seven modules (Fig. 3, node border colors) for the predicted network. The proteins detected by the yeast two-hybrid analysis, which we added later, were not considered and therefore are not contained in the modules. Based on these seven modules, we yield a modularity of about 0.57 (*p*-value= 1*e*−13) for the predicted network which is significantly larger than the median modularity of 0.32 for the random networks.

We considered the **transitivity** or the **clustering coefficient**, respectively. The transitivity of the predicted network was about 0.35 (*p*-value= 3*e*−42), in contrast to the random networks’ median of transitivities of about 0.12. This is in accordance with the observation that small-world networks exhibit higher clustering coefficients or transitivities, respectively [44].

Subsequently, we considered the nodes’ **degree** distributions and the nodes’ **betweennesses** (see Additional file 11). While the betweenness distribution of the predicted network significantly differs from the betweenness distribution of the random networks (*p*-value= 1.17*e*−08), the significance test of the degree distribution did not resulted in such a significant *p*-value (0.09). Most probably this was due to one major drawback of the degree distributions. In contrast to the betweennesses, the degree distributions consists of only a few different discrete values, i.e. 13 for the predicted network and 17 for all random networks. Hence, the Kolmogorov-Smirnoff test was not able to significantly distinguish both distributions. To overcome this drawback it was more feasible to visually compare the courses of the random and the real degree distributions. As expected, the distributions of the node degrees of the randomly chosen interaction partners were bell-shaped (Additional file 10: Figure S2a), in contrast to the degree distribution of the predicted PPI network which rather follows a power-law distribution depicted in the log-log-scaled plot with the fitted regression line (Additional file 10: Figure S2b-bottom). The power-law distribution indicates that there are many nodes with a low degree and some nodes with a high degree (scale-free property). These hub nodes indicate that the corresponding proteins are probably major players within the pathway (see also Table 2). We combined the node degrees with the node betweennesses to get the top ten of the most important proteins in the network (Table 2). In addition, for each of these ten proteins, we used the *Saccharomyces* Genome Database [7] to achieve information about the effects on yeast strains where the corresponding homologous gene had been knocked out.

**Table 2:** The ten most important proteins of the predicted PPI network

Module no.	Protein	Alias (no.	Ranks	Total	Effects in
(color code)	identifier	of interactions)	degree /	rank	knockout
			between.		yeast strains
3 (turquois)	Pa_3_5250	PaATG8 (22)	1 / 1	2	ne, aa, dv
2 (green)	Pa_5_5550	PaATG9 (13)	2 / 4	6	ne, aa, dv
1 (red)	Pa_4_9630	PaTOR (11)	4 / 2	6	TOR1: ne; TOR2: e
2 (green)	Pa_1_20610	PaATG3 (13)	3 / 6	9	ne, aa, dv
6 (ocher)	Pa_3_7690	PaYPT1 (9)	9 / 3	12	e
2 (green)	Pa_1_14210	PaATG11 (9)	8 / 8	16	ne, ma
3 (turquois)	Pa_4_7460	PaATG12 (10)	5 / 12	17	ne, aa, dv
6 (ocher)	Pa_5_4470	PaTRS130 (10)	6 / 13	19	e
2 (green)	Pa_5_5670	PaSEC24 (7)	16 / 9	25	e
2 (green)	Pa_7_10890	PaATG1 (6)	22 / 5	27	ne, aa, dv

The first column gives the module and its color according to Fig. 3. The column “Ranks” gives the ranks based on the degree and the betweenness. We summed up both values to a total rank and sort the table in descending order. The last column gives the most important effects on the yeast strain where the corresponding homologous gene had been knocked out [7]. Abbreviations: ne=non-essential, aa=autophagy absent, dv=decreased viability, e=essential, ma=mitophagy absent. For PaTOR, two homologs exist, TOR1 and TOR2. The protein identifiers were adopted from the *P. anserina* genome database [10]

We describe these major players in more detail in the “Discussion” section.

### Protein coding genes of the autophagy PPI network are significantly often co-expressed during aging

We were interested in how the coding genes of the predicted proteins are connected on the gene regulatory level. Hence, we considered the former longitudinal transcriptome analysis [34] and computed the Pearson correlation coefficients of each pair of the corresponding age-dependent expression profiles. Applying a threshold of 0.9 for the Pcc, we found 165 co-expressed gene pairs among the autophagy-related genes (see Additional file 12). Gene pairs, which show similar expression profiles, are probably co-regulated during aging. We computed an expectation value of about 85 co-expressed gene pairs and a *p*-value of 1.008*e*−14, i.e. exhibiting 165 co-expressed gene pairs is very unlikely to occur by chance. Subsequently, we applied a threshold of −0.9 to search for autophagy-related gene pairs with mirrored expression profiles. The corresponding genes were probably contrarily co-expressed during aging. We found 54 pairs to be co-expressed in an opposite manner with an expectation value of 25 pairs and a *p*-value of 5.79*e*−07. Nevertheless, when comparing the co-expressed gene pairs with the PPI network, we did not find a significant overlap, i.e. if two proteins are interacting, then the corresponding gene pairs are not necessarily co-expressed. This finding suggests that autophagy is indeed strongly age-dependently regulated, but the regulatory program on the genetic level is different to that on the protein level.

Further, we tested whether the genes, coding for the proteins of the predicted PPI network, were more frequently up-regulated during aging than expected by chance as found in the former transcriptome study. We computed a *p*-value of 0.003728 indicating a significant number of up-regulated genes for a given confidence level of 0.99.

Overall, the additional consideration of the transcriptome data associated with the proteins of the autophagy PPI network led to two major conclusions. First, we found a significant number of co-expressed gene pairs and groups, suggesting a strong, age-dependent regulatory relationship of the predicted proteins on the gene expression level. Second, we confirmed that genes associated with autophagy are significantly up-regulated during aging. Figure 3 may appear contradictory to this result as it seems that there are more down-regulated than up-regulated expression profiles. In fact, in the transcriptome study we found three times more decreasing than increasing expression profiles. Hence, within the autophagy pathway there are significantly more increasing expression profiles than expected by chance given the ratio of down- to up-regulated profiles.

These findings substantiated that investigations on the autophagy pathway and its relation to aging should include both protein as well as gene expression data even if both biological levels are not comparable.

## Discussion

### Evidence for a biological network

The presented PPI network of autophagy in *P. anserina* is based on a computational prediction approach combined with additional interactions deduced from the yeast two-hybrid analysis. We applied well-established, mathematical approaches to verify that the network was not generated by chance. We considered topological features which are known to be valid for biological networks.

One of these features is the larger network diameter in contrast to random networks, which is due to the higher degree of modularization. The second feature is the cluster coefficient, which is higher than its random counterpart. A high cluster coefficient indicates that some highly connected subgraphs exist. Often in a biological network, subgraphs can be interpreted as functional sub-pathways, for example as a metabolic or signaling pathway.

The autophagy pathway can be manually divided into the seven disjunctive sub-pathways: 1. induction and initiation, 2. targeting of substrates, 3. autophagosome nucleation, 4. autophagosome expansion and completion, 5. autophagosomal fusion with the vacuole, 6. the breakdown of the autophagosomes and the substrates, and 7. export of the secreted components.

Strikingly, also the independent module detection approach proposed seven modules of highly connected autophagy protein groups. The presence of important marker proteins led to the following alignment: one module which comprises the TOR kinase (red module in Fig. 3) corresponded to the initiation processes. Another module referred to the induction process, involving the putative proteins PaATG1, PaATG13, PaATG11, PaVAC8, PaATG17, and other proteins necessary for autophagy induction after signaling by the TOR pathway (green module in Fig. 3).

A third module covers the autophagosome nucleation and / or elongation step, comprising the proteins PaATG5, PaATG7, PaATG8, and PaATG12 (turquois module in Fig. 3).

We considered the degree distribution and the betweenness, two topological features which provide information about “major players” in the network. The degree distribution of the predicted network reveals that there are many nodes with a small number of neighbors, but a few nodes with much more interacting partners. This is in accordance with the scale-free property of “real-world” biochemical networks.

### Hub nodes and evolutionary conserved interactions

The interactions of the yeast two-hybrid analysis were not included in the topological analysis. In contrast to the prediction approach, where we considered all possibilities of interactions, the yeast two-hybrid analysis aimed to reveal interaction partners of only PaATG8 and was not statistically independent. Table 2 lists the ten most important proteins in the PPI network based on their degrees and betweennesses. In addition, for these ten proteins we included the phenotype information of the corresponding knockout yeast strains which is provided by the *Saccharomyces* Genome Database [7]. Since, for most yeast proteins the corresponding knockout strains are available, we were interested in how a loss of the homologous proteins affects the organism. Interestingly, eight of these proteins are either essential or a loss leads to a decreased viability and, more importantly, to an absence of autophagy. The two exceptions are, first, the ATG11 yeast mutant which exhibits a loss of mitophagy and second, PaTOR. Since, for PaTOR two homologs exist in yeast, TOR1 and TOR2, we included the information of both phenotypes. We found that in contrast to *Tor1*, *Tor2* is an essential gene. These findings reinforced the assumption that a high node degree generally correlates to the node’s importance for the pathway.

On the very top of the list of the ten most important proteins, we found the protein **PaATG8**, which is involved in most interactions (highest degree) and also in many of the shortest paths (highest betweenness). The finding of PaATG8 to be one of the major hub proteins in autophagy corresponds to its known relevance for the autophagy pathway in other organisms. The autophagy-related protein PaATG8 is homologous to the yeast ATG8 protein and the LC3 protein in mammals. The ATG8 and LC3 proteins are known to be essential for formation and expansion of the autophagosome membrane during autophagy and to be furthermore crucial for other autophagy-related processes, such as cargo delivery into autophagosomes or for selective targeting of cytosolic components for lysosomal and / or vacuolar degradation [38]. Due to its ubiquitin-like function, the ATG8 protein is part of a larger protein complex which binds to the phospholipid membrane of the autophagosome. In addition, it binds different cargo receptors during selective autophagy and mediates the transport of the autophagosomes to the lysosome or the vacuole, respectively. Due to this known relevance for autophagy, we initially applied the yeast two-hybrid analysis to reveal putative interaction partners of PaATG8 in *P. anserina*, before we started the autophagy interaction study. For most of the proteins identified in the yeast two-hybrid analysis, we found only a weak evidence for interaction with PaATG8, because most of them were identified only in one or two clones (see Additional file 10: Figure S1). These proteins may represent experimental artefacts and unspecific binding issues. They have not been predicted by the bioinformatics approach, indicating that they have not been found and probably do not exist in other species. In contrast, the two most promising interacting partners, **PaNBR1** and **PaATG4**, for which multiple independent clones (26 and 7, respectively) were selected in the yeast two-hybrid screen, were also found in the predicted PPI network.

In mammals, the **NBR1** protein contains a LIR-motif (LC3-interaction region) enabling the proteins to bind to LC3 (aPaATG8 homolog). At the C terminus of PaNBR1, a putative LIR-motif was found with the LIR consensus sequence [DE]-[DE]-[DE]-[WFY]-X-X-[LIV] [15] probably responsible for interaction with PaATG8. Kraft et al. (2010) [20] describe that NBR1 mediates autophagosomal engulfment of misfolded protein aggregates which were too large to get degraded by the 26S proteasome. Interestingly, the number of transcripts of the *P. anserina* PaNBR1 increases during aging by the factor of 3.1. This could be due to the increasing number of misfolded and larger protein aggregates during aging. In the same transcriptome study we found that the expression of proteasomal genes significantly decreases during aging. We hypothesize that in *P. anserina* during aging, the increased number of *PaNbr1* transcripts also leads to an increased number of PaNBR1 proteins to prevent an accumulation of protein aggregates due to the declined function of the proteasome system.

The protein **PaATG4** is another PaATG8-interacting protein identified by the prediction and the yeast two-hybrid analysis. In yeast and other organisms, ATG4 together with **ATG7** and **ATG3** is active in the conjugation of phosphatidylethanolamine with ATG8, enabling the association of ATG8 with the pre-autophagosomal structure. Remarkably, all three interactions, ATG8 with ATG3, ATG4, and ATG7, were found in both reference species (see purple edges in Fig. 3), indicating an evolutionary conservation of this conjugation system already proposed by Kikuma and Kitamoto (2011) [17] for *Aspergillus oryzae*. In this filamentous fungus, it was found that the deletion of *AoAtg4* leads to impaired autophagy. The same was observed in *Sordaria macrospora*, another filamentous fungus, after transcriptional down-regulation of the ATG7 homolog [29]. The third protein is the E2 enzyme **PaATG3** whose homologs are very important for autophagy in other organisms [1, 25]. It is on the very top of the list in Table 2, indicating its importance for the predicted autophagy PPI network. Summarizing, we suggest that PaATG8 and its interactions with the conjugation system, consisting of **PaATG7**, **PaATG4**, and **PaATG3**, play a crucial role in the autophagy process in *P. anserina* as well.

The ubiquitin-like protein **PaATG12** is the fourth protein which is involved in putative evolutionary conserved interactions (purple edges in Fig. 3). It is associated with PaATG8 and the proteins forming the conjugation system. The yeast homolog, ATG12p, is activated by **ATG7** and finally linked to **ATG5** to build the ATG12p conjugation system, which is essential for autophagosome formation [29]. Meijer et al. (2007) [24] described that genes, coding for the corresponding proteins or interacting partners, respectively, are also conserved in filamentous fungi.

Three additional proteins and interactions were predicted to be evolutionary conserved, since they are based on proteins and interactions in human and in yeast. These are the two vacuolar protein-sorting enzymes, **PaVPS15** and **PaVPS34**, and the autophagy-related protein, **PaATG6**. In *S. macrospora*, it was not possible to generate knockout strains for these genes, suggesting that the corresponding proteins are essential for viability in this fungus [40]. Since both are very homologous to their counterparts, PaVPS15 and PaVPS34 (with a BLAST E-value of 0 each), it is likely that they have similar effects on viability in *P. anserina*. The evolutionary conservation and importance of ATG6 and corresponding homologs (like the mammalian Beclin 1) for autophagy was already described for various other organisms. Even its relation to aging processes and lifespan control has already been discussed [6, 13, 26].

The second and third most important proteins in the predicted network, **PaATG9** and **PaTOR**, were equally important. The reason is that we considered the autophagy pathway, but not all the other biological processes which are affected by the TOR kinase. Nevertheless, for autophagy in *P. anserina*, PaATG9 seems to play an essential role. For other systems, it is known that ATG9 is activated by **ATG1** which is also in the list of the ten most important proteins. The activated ATG9 is required for recruitment of ATG8 to the pre-autophagosomal structure and therefore essential for proper efficiency of autophagy [31].

### Transcriptional relationship of aging and autophagy

Although the current study is mainly focused on the PPI network of autophagy in *P. anserina*, we also considered the related transcripts and corresponding expression profiles in the course of aging due to two considerations. First, we were interested in a holistic and systems biology view of autophagy and its relationship to aging. Since we hold a comprehensive set of gene expression data, we included the data into the network. Second, we presumed that there is a considerable relationship of transcription, protein level, and activity of autophagy, because autophagy-associated gene expression significantly increases during aging [34], and the subsequent experimental validation identified an age-related increase of autophagy in later stages of the life cycle, before the system finally breaks down [19].

Initially, we were interested in the general expressional behavior and analyzed whether the genes, coding for the predicted proteins, are more often co-expressed than expected by chance. We found 165 co-expressed gene pairs with a *p*-value of 1.008*e*−14, indicating a very significant and strong co-expression within the set of autophagy-related genes. That are approximately twice as many co-expressed gene pairs as expected in a random network. To the best of our knowledge, it is the first time that a significant degree of co-expression during aging of autophagy-associated genes is demonstrated, supporting again the relevance of the autophagy pathway for aging processes in *P. anserina*. This approach revealed that there is a significant number of co-expression within the genes, which correspond to the predicted proteins and it was experimentally validated that the predicted components of the network are strongly related with each other. By means of the topological analysis, we verified that the predicted interactions exhibit properties which are characteristic for biological networks. Next, we analyzed the age-dependent expressional tendency of each single gene. At a first glance, it seems that the majority of transcripts, coding for the proteins of the autophagy PPI network, were down-regulated during aging what would be in contrast to the former transcriptome study [34]. Nevertheless in that study, there were essentially more down-regulated than up-regulated transcripts, which was probably due to a general and continuous decline of the organism during aging. Hence, it was of great importance to statistically prove whether the number of up-regulated transcripts exceeds the expectation. Indeed, the *p*-value of 0.0037 indicated that the amount of ten transcripts, showing a continuously up-regulation during aging, was more than expected by chance, which is in accordance with the transcriptome study.

Since up-regulation of autophagy seems to act as an important mechanism and rescue effort during aging in *P. anserina*, we wanted to identify the single up-regulated components of autophagy. In addition to the hub proteins, we analyzed and discussed the ten proteins, for which the corresponding transcripts exhibit continuously up-regulated expression profiles during aging. These proteins are listed in Table 3 and marked with red arrows in Fig. 3. In the following, we refer **p**roteins with **u**p-**r**egulated expression profiles of the corresponding genes as **PUR** proteins. Interestingly, only one of the PUR proteins is also a hub protein, PaTRS130. In yeast, the homologous protein, Trs130, is part of the TRAPP II complex (Transport protein particle II), which mediates the trafficking of autophagy proteins from the Golgi to pre-autophagosome structures. It was found that autophagy was impaired in a trs130 temperature-sensitive mutant, suggesting an important role for the early autophagy induction processes [45].

**Table 3:** Proteins with up-regulated expression profiles (PUR proteins)

Protein	Alias	Module no.	Pearson correlation	Ratio
Pa_0_1500	PaATG13	2	0.87	1.74
Pa_1_10350	PaMAN1	2	0.87	2.10
Pa_5_4470	PaTRS130	6	0.87	3.94
Pa_7_7880	PaIRS4	1	0.86	2.79
Pa_1_11020	PaATG6	7	0.84	3.48
Pa_1_5330	PaRPD3a	2	0.81	1.65
Pa_4_2219	Pa_4_2219	TH	0.79	4.96
Pa_2_12420	PaNBR1	3	0.78	3.13
Pa_6_1830	PaVPS15	7	0.77	2.40
Pa_2_2450	Pa_2_2450	TH	0.72	3.38

For ten proteins, the corresponding transcripts were up-regulated during aging (see expression profiles in Fig. 3 of the nodes marked by red arrows). The higher the Pearson correlation coefficient the straighter increases the related expression profile or the number of gene products, respectively. TH indicates that the protein was not assigned to any module, since it was only found in the yeast two-hybrid analysis. The last column gives the ratio of the transcript abundance from the first to the last day of measurement. The protein identifiers were adopted from the *P. anserina* genome database

The other PUR proteins are not important hub nodes with a greater number of interactions or crossing shortest paths. This can be explained by the assumption that gene expression is not or only weakly related with the actual relevance and importance of the corresponding protein. Because we suppose a considerable relationship of transcription, protein level, and activity of autophagy, we also assume that these PUR proteins may play important regulatory roles for down-streamed sub-pathways. For example, we previously supposed that the increase of the transcripts of PaNBR1 is caused by an increased number of misfolded and larger protein aggregates. Another example is the PaATG13 protein, which is involved at the very beginning of the autophagy process when it becomes activated or deactivated by the TOR kinase. Nevertheless, the two most promising PUR proteins are PaATG6 and PaVPS15, since both are the only ones which are involved in evolutionary conserved interactions. Furthermore, PaATG6 homologs were already described to be relevant for aging processes in other organisms. It will be of interest to see whether the corresponding transcripts show an increased abundance during aging in other systems.

Summarizing, the provided PPI network of autophagy in *P. anserina* is based on experimental data and mainly on a prediction approach which relies on sequence homology. Since there are known and reasonable drawbacks of such prediction methods [42], it is important to carefully analyze the results by appropriate mathematical approaches. Finally, only biological experiments allow the validation of the drawn conclusions. Nevertheless, we substantiate the reliability of the predicted network using two main approaches. First, we applied different analyses which revealed topological features that are significant for biological networks. Since these topological features were probably already effective for the origin networks of yeast and human, they indicate that the transfer of the interactions relies on well-defined rules and assumptions and thus, no random network was developed. Second and in addition to the first assumption, the experimentally achieved expression data indicated that the predicted components are strongly related and with high probability belong to the same biological pathway. Hence, we provide a statistically validated biological network, which also was partly substantiated by experimental data and which indeed has to be finally confirmed by subsequent experiments. The network can help to plan further studies which aim to investigate the relationship of aging and autophagy in *P. anserina*.

## Conclusions

In this study, we developed and investigated the putative protein-protein interaction network of autophagy in the aging model *P. anserina*. The study is based on the PPI networks of the reference organisms, human and yeast, and own experimental data. Using well-defined and established network analysis approaches, we demonstrate that the present PPI network is not random, but a “real-world” one. For expansion of the predicted network, we integrated the data of a yeast two-hybrid analysis. Additionally, we considered age-dependent gene expression data from a former transcriptome study to expand and combine the current knowledge of autophagy in *P. anserina* to one single unifying biological network.

We identified hub proteins that act as major players, e.g. PaATG8, PaATG9, and PaATG3, and seven functional modules, representing sub-pathways within autophagy. We confirmed that genes associated with autophagy are significantly more frequently up-regulated during aging than expected by chance. In general, autophagy-associated genes are much more co-expressed and, therefore, probably more co-regulated than randomly chosen gene groups.

In contrast to other model organisms, only little was known about PPIs of autophagy in *P. anserina*. Only the basal components of autophagy in *P. anserina* had been investigated until now. The present study aimed to collect, combine, and compile already existing data from *P. anserina* as well as from other organisms to achieve a new and general overview on autophagy in *P. anserina* which has not been available before. Moreover, since *P. anserina* is a well established aging model the provided network allows further investigations to gain a deeper knowledge on autophagy during aging of *P. anserina* as well as of other organisms.

## Additional files

Additional file 1 Nucleotide sequence of the vector pLexA-DIR. The sequence is provided as text-based FASTA file. (FASTA 6 kb)

Additional file 2 Nucleotide sequence of the vector pGAD-HA. The sequence is provided as text-based FASTA file. (FASTA 8 kb)

Additional file 3 Yeast two-hybrid analysis. The results of the yeast two-hybrid analysis including the accession ID, the number of clones, the trivial names, and the putative function. For each prey clone of the non-singletons the position of the first amino acid is given in a separate sheet. In addition, the file contains each edge and node of the hybrid network, which was deduced from the interactions of the yeast two-hybrid analysis and the corresponding homologous proteins from yeast and human (see Additional file 10: Figure S1). Two edge types can be distinguished. First, edges indicating an interaction, where the source and the target node correspond to proteins in the same species. Second, edges indicating a homologous relation, where the source protein belongs to *P. anserina* and the target protein to yeast or human. UniProt accession numbers were used as identifiers for proteins of yeast and human. The identifiers for *P. anserina* proteins were adopted from the *P. anserina* genome database [10]. Species were distinguished by their NCBI taxonomy IDs: *Homo sapiens* (9606), *Podospora anserina* (5145) and *Saccharomyces* *cerevisiae S288c* (559292). (XLS 88 kb)

Additional file 4 *P. anserina* autophagy pathway from KEGG. List of all proteins and interactions for the autophagy pathway in *P. anserina* provided by the KEGG database. The identifiers for *P. anserina* proteins are either KEGG accession numbers (PODANSxxx) or were adopted from the *P. anserina* genome database (Pa_xx_xx). (XLS 37 kb)

Additional file 5 Results and statistic of topological and random network analysis. The file contains all topological values of each randomly generated network. In addition, the results of the comparison of the predicted network with the random networks are provided. (XLS 1896 kb)

Additional file 6 Lists of reference proteins and interactions. The file contains each protein and interaction from both reference species, which initially had been used for network prediction. UniProt accession numbers were used as identifiers for proteins of yeast and human. (XLS 133 kb)

Additional file 7 Parameter fitting. The results of the parameter fitting approach are given, including the lower and upper bounds of the E-values, their logarithms to the base 10, the differences between the logarithmic upper and lower bounds as well as the number of proteins and interactions in the reference KEGG network and in the predicted network. The parameters we have applied for the prediction approach in Path2PPI are highlighted red. (XLS 43 kb)

Additional file 8 Hybrid PPI network. This file gives the underlying data of Fig. 2. In contrast to Additional file 1, this file contains all interactions, proteins and homologous relations of the autophagy hybrid PPI network, comprising both reference PPI networks, the predicted PPI network and the homology network which connects all three PPI networks. As for Additional file 1, two edge types can be distinguished. First, edges indicating an interaction, where the source and the target node correspond to proteins in the same species. Second, edges indicating a homologous relation where the source protein belongs to *P. anserina* and the target protein to yeast or human. UniProt accession numbers were used as identifiers for proteins of yeast and human. The identifiers for *P. anserina* proteins were adopted from the *P. anserina* genome database. Species were distinguished by their NCBI taxonomy IDs: *Homo sapiens* (9606), *Podospora anserina* (5145) and *Saccharomyces cerevisiae S288c* (559292). (XLS 105 kb)

Additional file 9 Autophagy PPI network of P. anserina. This files contains the tabular representation of Fig. 3 and provides all underlying data of the constructed PPI network of autophagy. The network consists of all interactions from the prediction approach and the yeast two-hybrid analysis. It includes the ID of the interaction, the source ID, the target ID, and the score. This file also contains further information about the corresponding reference interactions. For each predicted interaction, the reference interaction from yeast and / or human with the corresponding publication, author, and interaction type is listed. In addition, the file contains the expression data which are depicted as expression profiles in Fig. 3. UniProt accession numbers were used as identifiers for proteins of yeast and human. The identifiers for *P. anserina* proteins were adopted from the *P. anserina* genome database. Species were distinguished by their NCBI taxonomy IDs: *Homo sapiens* (9606), *Podospora anserina* (5145) and *Saccharomyces cerevisiae S288c* (559292). (XLS 271 kb)

Additional file 10 Additional figures. The figures concern the graphical representation of the results of the yeast two-hybrid analysis and node degree distributions of the random network and of the predicted network. (PDF 243 kb)

Additional file 11 Node degrees and betweennesses. The degrees and betweennesses for each node of the predicted autophagy PPI network, including the IDs, the alias names, the node degrees, the betweenesses, the rank of the degrees, the rank of the betweenesses, the rank of the sum and the corresponding module number. The identifiers for *P. anserina* proteins were adopted from the *P. anserina* genome database. (XLS 43 kb)

Additional file 12 Co-expressed genes. All co-expressed gene pairs deduced from the predicted PPI network and the former longitudinal transcriptome analysis, including the IDs of the genes and the Pearson correlation coefficient. The identifiers for *P. anserina* proteins were adopted from the *P. anserina* genome database. (XLS 48 kb)

## Abbreviations

ATG: Autophagy-related genes
BLAST: Basic local alignment search tool
cDNA: Complementary deoxyribonucleic acid
GO: Gene ontology
Pcc: Pearson correlation coefficient
PPI: Protein-protein interaction
PUR: Proteins with up-regulated expression profiles of the corresponding genes
QC: Quality control
