# Supplementary material for: The autophagy interaction network of the aging model Podospora anserina
Source: BMC Bioinformatics. 2017 Mar 27;18:196. doi: 10.1186/s12859-017-1603-2 (PMC5369006; doi:10.1186/s12859-017-1603-2)
Supplement: Supplementary file 10 — Additional figures. The figures concern the graphical representation of the results of the yeast two-hybrid analysis and node degree distributions of the random network and of the predicted network. (PDF 243 kb) [file 12859_2017_1603_MOESM10_ESM.pdf]

Additional figures

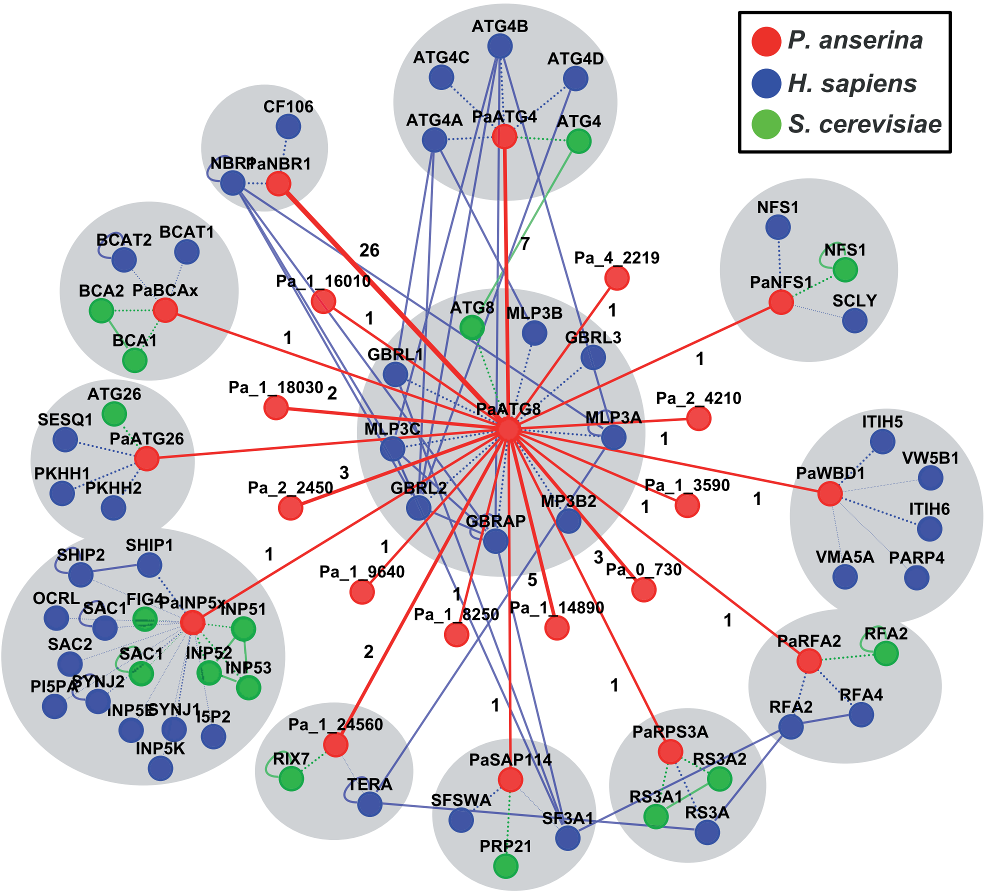

Figure S1: Graphical representation of the yeast two-hybrid analysis. The image gives each putative interaction partner of PaATG8 (red nodes and red edges) and the homologs to these proteins and PaATG8, which fulfill the criteria for homology of the former prediction approach. Each of these "homology groups" are surroundeded with gray circles, i.e. for PaATG4, there were four homologs in human and one in yeast. The numbers at the edges indicate the number of clones which were found by the yeast two-hybrid analysis. The pale blue and green edges give all known interactions of the homologous proteins provided by the corresponding iRefIndex files of human (blue) and yeast (green). The corresponding lists can be found in Additional file 3.

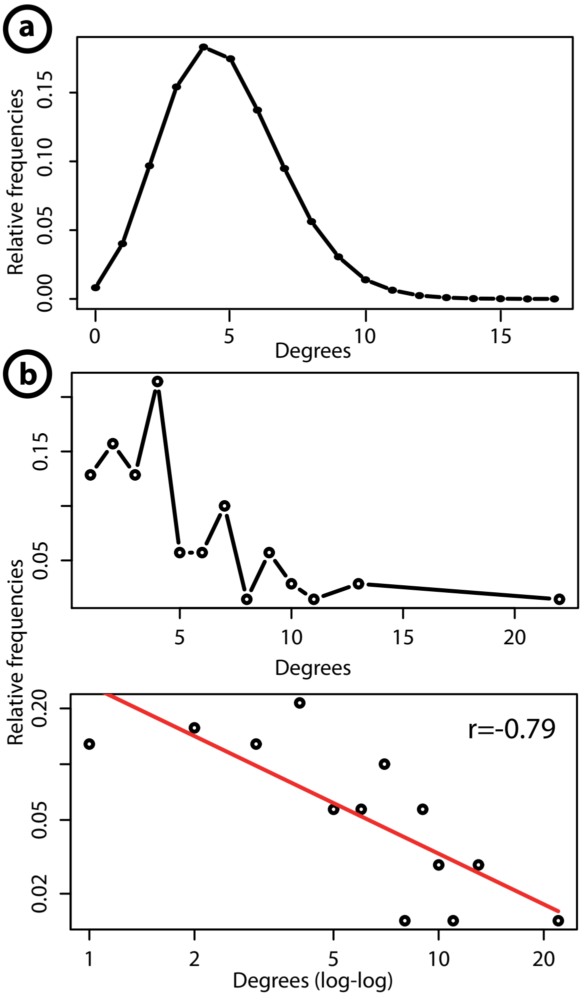

Figure S2: Node distributions. **(a)** The node distributions of random networks. **(b)** The node distribution of the predicted PPI network (top) and as log-log-scaled view (bottom). The fitted linear function with a regression value of 0.79 indicates that the distribution follows a power law which is characteristic for biological networks. This scale-free property means that some nodes are highly connected and many nodes are weakly connected to other nodes.
